# NRXN1 depletion in the medial prefrontal cortex induces anxiety-like behaviors and abnormal social phenotypes along with impaired neurite outgrowth in rat

**DOI:** 10.1186/s11689-022-09471-9

**Published:** 2023-02-03

**Authors:** Di Wu, Jiansheng Zhu, Lianghui You, Jingyu Wang, Sufen Zhang, Zhonghui Liu, Qu Xu, Xiaojie Yuan, Lei Yang, Wei Wang, Meiling Tong, Qin Hong, Xia Chi

**Affiliations:** 1 Department of Child Healthcare, Women’s Hospital of Nanjing Medical University, Nanjing Maternity and Child Health Care Hospital, Nanjing, China; 2 The Fourth Affiliated Hospital of Nanjing Medical University, Nanjing, China

**Keywords:** Neurodevelopmental disorders, NRXN1, Anxiety-like behavior, Social behavior, Neurite outgrowth, Cell adhesion molecules

## Abstract

**Background:**

Neurodevelopmental disorders (NDDs) are a group of disorders induced by abnormal brain developmental processes. The prefrontal cortex (PFC) plays an essential role in executive function, and its role in NDDs has been reported. NDDs are associated with high-risk gene mutations and share partially overlapping genetic abnormalities.

**Methods:**

Neurexins (NRXNs) are related to autism spectrum disorder (ASD) and attention-deficit hyperactivity disorder (ADHD). NRXN1, an essential susceptibility gene for NDDs, has been reported to be associated with NDDs. However, little is known about its key role in NDDs.

**Results:**

NRXN1 downregulation in the medial PFC induced anxiety-like behaviors and abnormal social phenotypes with impaired neurite outgrowth in Sh-NRXN1 in prefrontal neurons. Moreover, tandem mass tag (TMT)-based proteomic analysis of rat brain samples showed that NRXN1 downregulation led to significant proteome alterations, including pathways related to the extracellular matrix, cell membrane, and morphologic change. Furthermore, full-automatic immunoblotting analysis verified the differently expressed proteins related to cell morphology and membrane structure.

**Conclusions:**

Our results confirmed the association of NRXN1 with abnormal behaviors in NDDs and provided richer insights into specific prefrontal knockdown in adolescence, potentially expanding the NRXN1 interactome and contributing to human health.

**Graphical abstract:**

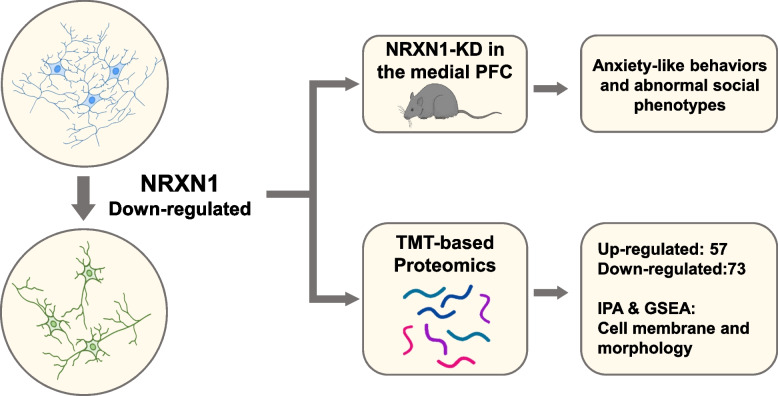

**Supplementary Information:**

The online version contains supplementary material available at 10.1186/s11689-022-09471-9.

## Highlights

- NRXN1 depletion induced anxiety-like behaviors and abnormal social phenotypes in rats.
- Knockdown of NRXN1 impaired neurite outgrowth in primary neurons.
- Downregulation of NRXN1 might downregulate the proteins associated with cell morphology and membrane structure

**Supplementary Information:**

The online version contains supplementary material available at 10.1186/s11689-022-09471-9.

## Introduction

Neurodevelopmental disorders (NDDs) are a diverse group of chronic disorders presented as development and function impairments. Although NDDs manifest early in development and are recognized childhood disabilities, they could persist across a lifespan, severely affecting the quality of life [1]. NDDs include intellectual disability (ID), autism spectrum disorder (ASD), attention-deficit hyperactivity disorder (ADHD), communication disorders, motor disorders, and specific learning disorders [2]. The clinical presentation of NDDs is heterogeneous and often manifests as complex patterns of impaired cognition, communication, adaptive behavior, and psychomotor ability. For instance, the core symptoms of ASD are behavioral abnormalities, including difficulty in social interaction and communication and restrictive and repetitive behaviors [3]. Individuals with ADHD manifest symptoms of inattention, hyperactivity, and impulsivity. It has long been recognized that the etiology of NDDs is complicated and heterogeneous [4–7]. Thus, it is essential to identify the underlying genetic events in NDDs.

Recent research has shown that NDDs may share partially overlapping genetic abnormalities [6, 8, 9]. Several candidate genes involved in neural structure and function were investigated. Multiple studies suggest that neurexins (NRXNs) are associated with ASD and ADHD [10–12]. NRXN family plays a core role in synapse organization and transmission. Missler and colleagues generated mouse models by deleting the first exons of the *α*-neurexin. Nonetheless, the mice with triple *α*-neurexin KOs died soon after birth, proving that a minimum of two intact *α*-neurexin are needed for survival [13].

Human genetic studies revealed that the mutation and transcriptomic/splicing dysregulation of *NRXNs*, predominantly NRXN1, is one of the most significant risk factors for a broad spectrum of neurodevelopmental, psychiatric, and neuropsychological disorders [14]. NRXN1 gene expresses two primary transcripts from two independent promoters to yield longer (*α*) and shorter (*β*) proteins. Two classic autistic behavior, an increase in repetitive grooming and significant changes in motor learning abilities, were observed by Etherton and colleagues in *α*-neurexin-1 KO mice with a hybrid SV129/C57BL/6 background, despite no changes in their social behaviors experiments [15]. Grayton et al. observed high anxiety levels and altered social approach without any loss of working memory in *α*-neurexin-1 KO mice with a different background (pure C57BL/6J strain) [16]. The NRXN1 deletions in ASD and schizophrenia (SCZ) tend to be commonly heterozygous [17]. While the impact on sociability and the anxiety-like behavior appears limited, the NRXN1α^+/−^ mice also displayed social memory deficiencies. It has long been recognized that the etiology of NDDs is complicated and heterogeneous [18, 19]. So far, only minimal work has been done using the NRXN1 heterozygous (HET) mice. Prefrontal cortex (PFC) plays a significant role in executive functions, including cognitive flexibility and attention [20]. The deletion of NRXN1 dramatically increases the risk of NDDs [21, 22]. The metabolism of the PFC region is altered by NRXN1α deletion, as suggested by the available evidence. Besides, the heterozygosity of *α*-neurexin-1 also reduced the efficiency of the functional brain networks. The PFC may be dysfunctional in humans from the clinical cases reported with 2p16.3 deletions [21]. In humans and rodents, the PFC plays an essential role in social cognition and emotional regulation [20]. We examined the behavioral performance in PFC NRXN1 knockdown rats and sought to quantify phenotypes relevant to human disorders such as impaired social interaction and repetitive behavior.

NRXN1 has been recognized as an essential susceptibility gene for NDDs. However, although the NRXN1 role during synaptogenesis has been extensively studied, there its role in neurogenesis remains controversial. Changhui Pak and colleagues found that morphological properties, including total neurite length, number of primary processes, and neurite branch points were unchanged in NRXN1 mutant human neurons [23]. On the contrary, Erin Flaherty and colleagues showed decreased neurite number and total length in NRXN1^+/−^ hiPSC-neurons [24]. Therefore, it is important to unveil the pathogenicity of NRXN1 in aberrant behaviors, such as locomotor activity and social behavior [25–27]. M. V. Pletnikov showed that decreased neurite complexity is associated with spontaneous locomotor activity and social behaviors [28]. Our recent research confirmed that NRXN1 orchestrates a transsynaptic network and is responsible for impaired learning and memory in ADHD [29].

This study observed anxiety-like and alerted social behaviors in PFC NRXN1 knockdown junior rat models with impaired neural processes in Sh-NRXN1 neurons in primary neurons. Moreover, TMT-based proteomic analysis displayed that NRXN1 depletion caused significant expression alterations in 130 proteins associated with the extracellular matrix, cell membrane, and morphologic change. Full-automatic immunoblotting analysis was performed to verify the differently expressed proteins identified by mass spectrometry.

Our findings confirm that NRXN1 deletion is highly correlated with social behavior, stereotyped behavior, and anxiety, which were highly consistent with previously reported results. These behavioral changes give additional insight into the PFC dependence of NRXN1-specific knockdown. Our results elucidated the underlying molecular mechanisms of NRXN1 in NDDs and expanded the NRXN1 interactome.

## Materials and methods

### Chemicals and reagents

The mouse antibodies used were as follows: anti-NeuN (Ab104225, Abcam, dilution IF: 1:500). Rabbit antibodies used in this study were as follows: anti-NRXN1 antibody was used (GTX54845, GeneTex, dilution, IF:1:30, IHC:1:30; Santa Cruz, sc136001, WB:1:100), anti-MAP 2 (Ab32454, Abcam, dilution IF: 1:500), anti-GFP (AF5066, Beyotime, dilution IF: 1:100), and anti-GAPDH (10494, Proteintech, dilution WB 1:1000). Other antibodies were as follows: Donkey polyclonal Secondary Antibody to Mouse IgG (Alexa Fluor594, ab150108, Abcam, dilution IF 1:1000), Donkey polyclonal Secondary Antibody to Rabbit IgG (Alexa Fluor488, ab150061, Abcam), HRP-conjugated AffiniPure Goat Anti-Mouse IgG(H+L) (SA00001-1, Proteintech, dilution WB:1:5000), and HRP-conjugated AffiniPure Goat Anti-Rabbit IgG(H+L) (SA00001-2, Proteintech, dilution WB:1:5000).

### Animal housing and treatment

Male Sprague Dawley (SD) rats at the prepubertal age (3 weeks) were maintained in a standard-controlled environment at 23 ± 1 °C and relative humidity of 50 ± 5% on a dark/light cycle and obtained from Charles River, Beijing Vital River Laboratory Animal Technology Co. (Beijing, China). All animals had free access to water and food. The Animal Ethics Committee of Nanjing Medical University approved the research protocol (approval no. IACUC-1711004). Before injections, animals were acclimated for 1 week. At 4 weeks old, rats were anesthetized with 10% chloral hydrate (0.35 ml/100 g) by intraperitoneal injection. The depth of anesthesia was determined by monitoring reflexes, skin color, heart rate, and respiratory rate. PFC knockdown of NRXN1 was performed in anesthetized rats by placing them on a stereotactic instrument, followed by intracerebral injection of AAV9-NRXN1-GFP (positive-sense strand: AGAUGAGCUUCCAGCUGAAdTd, viral titer: 1 × 10^12 TU/ml) targeting exon one. AAV9-NRXN1-GFP was constructed by Hanbio Biotechnology Co. (Shanghai, China). Adeno-associated viral vector containing irrelevant sequences (AAV-Nc-GFP, Hanheng Company) was used as a negative control. Twenty-seven rats were randomly divided into three groups, wild type (*WT* = 9), Sh-Nc (*n* = 9), and Sh-NRXN1 (*n* = 9). AAVs were injected into PFC at a 20° angle (anteroposterior = 2.6 mm; mediolateral = ±0.7 mm to bregma; dorsoventral = −3.0 mm). The infusion was performed with a 10 μl volume at 1 μL/min with a 0.5-mm flat needle, and the syringe was maintained in place for 10 min after the infusion was completed. The syringe was withdrawn gradually to prevent overflow. Animals were monitored daily following surgery and given 2 weeks to allow for full expression of the viral constructs before behavioral testing. To verify injection sites, 30-mm-thick sections were cut at the level of PFC using a cryostat. AAV infection was verified by immunohistochemical analysis of the GFP vector. Animal experiments were performed following the approved guidelines and experimental protocol of Nanjing Medical University.

### Open-field test

For the open-field test (OFT), we used a black box with a bottom of 40 × 40 cm and a height of 45 cm with a 4-W cold light source above the center of the box. Animal activity was recorded using the Shanghai Jiliang Animal Behavior Analysis System (Shanghai, China). Behavioral tests were performed 2 weeks after the AAV injection. Rats were placed in the center of the open-field apparatus, and their activity under light conditions was recorded by an infrared camera system and video synthesizer. We used a computer analysis system to analyze movement trajectories. To make sure each rat’s performance did not affect the behavior the next, the box was cleaned with 75% alcohol solution after each experiment. Self-grooming behavior was also measured during OFT tests. Behavior was video-recorded for 10 min. Time spent in self-grooming and the number of bouts were manually recorded. Detailed descriptions were illustrated in the supplementary materials.

### Three-chamber sociability test

The three-chamber setup (120 cm × 80 cm × 40 cm) consisted of a black acrylic floor and transparent acrylic walls separating the arena into three small compartments. An opening to the outer compartments could be closed with removable slide doors on the walls of the inner compartment. Two cylinders with a diameter of 22 cm (40 cm in height) were placed in the outer compartments for the stimulus rats during testing. One cylinder contained an unfamiliar male SD rat (age 6 weeks; “Stranger”), whereas the other remained empty. These cylinders contained acrylic bars placed 15 mm apart to allow social contact while preventing aggressive assaults. All three-chamber experiments were performed in dim light conditions (10 lux). Animals were moved to the experimental room for environmental adaptation 1 day. Before the formal experiment, each experimental animal underwent a 15 min adaptation time. Time spent exploring either cage was measured for 10 min. This is considered a test of sociability. Rat activity was recorded using the Shanghai Jiliang Animal Behavior Analysis System (Shanghai, China). Detailed descriptions were illustrated in the supplementary materials.

### Primary neuronal culture

The Animal Ethics Committee of Nanjing Medical University approved all animal experiments. The PFC was dissected from SD rat embryos (E18), and cells were dissociated using trypsin and titrated through a Pasteur pipette. The neurons were plated on coverslips with poly-L-lysine in DMEM with 10% horse serum at a density of 1 × 10^5 cells/cm^2^. When neurons were attached to the coverslip within 24 h, the medium was changed to neurobasal media with a 2% B27 supplement.

### NRXN1 knockdown in vitro

Knockdown of NRXN1 in prefrontal neurons was performed by lentivirus infection (viral titer, 1 × 10^8 TU/ml; Hanbio Biotechnology Co., Shanghai, China). In a preliminary experiment, we infected the virus with GFP markers to determine infection efficiency, and neurons were infected at a multiplicity of infection (MOI) of 10. We used viruses without fluorescent labels in formal transfection experiments. Cells were divided into three groups, a blank control group (uninfected), an Nc group (transfected with negative control lentivirus), and a lentivirus-shRNA group (transfected with target Sh-NRXN1 lentiviral vectors). Cell media was replaced with fresh Neurobasal media with a 2% B27 supplement 12 h after transfection. Knockdown efficiency was determined by Western blot, PCR, and immunofluorescence after 72 h of infection.

### RNA isolation and quantitative real-time polymerase chain reaction (qRT-PCR)

Using the TRIzol reagent (Invitrogen, CA, USA) and the RNeasy Mini Kit (Qiagen GmbH, Hilden, Germany), the total RNA of the neurons was isolated using the TRIzol reagent (Invitrogen, CA, USA) and the RNeasy Mini Kit (Qiagen GmbH, Hilden, Germany) following the guidelines of the manufacturers. A NanoDrop 2000 system (NanoDrop Technologies, Wilmington, DE, USA) was applied to evaluate the concentration and purity of the RNA. A total of 1 μg RNA was reverse-transcribed into cDNA using the random hexamer primers, gDNA Eraser (RR047A; Perfect Real Time, Takara, Tokyo, Japan) from each sample and applying the PrimeScript™ Reverse Transcription (RT) reagent kit. The quantitative real-time PCR (qRT-PCR) was conducted on the Applied Biosciences ViiA 7 Real-Time PCR System (Invitrogen, Carlsbad, CA, USA) to measure mRNAs expression levels. The conditions were as follows: initial denaturation at 95 °C for 10 min, followed by 40 PCR cycles at 95 °C for 15 s, 60 °C for 30 s, and extension at 72 °C for 30 s, using the SYBR green method (Thermo Fisher Scientific Inc., Shanghai, China) by following the instructions of the manufacturer. The melting curve analysis was performed to confirm the selectivity of the PCR product, while the comparative cycle threshold (CT) method (2^−ΔΔCt^ method) was applied to determine the relative expression levels. The Basic Local Alignment Search Tool (BLAST) from the National Center for Biotechnology Information (NCBI) was consulted to corroborate the specificity of the amplified result, coupled with the designing of primers with Primer 5. Primers for qPCR are exon spanning. Table S1 illustrates the primer sequences in detail.

### Western blotting

Neurons (1 × 10^5 cells/cm^2^) were lysed in RIPA buffer (Beyotime, China). Protein extract quantification was performed using a BCA protein assay kit (Beyotime, China). Denatured protein samples were separated with 4–12% (GenScript, M00653) sodium dodecyl sulfate-polyacrylamide and then transferred onto the polyvinylidene difluoride membranes. Membranes were blocked with skim milk solution for 2 h followed by primary antibodies incubation at room temperature for 2 h. Primary antibodies for NRXN1 (Santa Cruz, sc136001) were diluted 1:100 in PBS-Tween-20. Primary antibodies for GAPDH (10494, Proteintech) were diluted at 1:5000. The membranes were washed thrice (about 10 min/wash) using Tween-20 (pH 8.0) and Tris-buffered saline (TBST). Subsequently, membranes were incubated at room temperature for 2 h, using the horseradish-peroxidase-conjugated secondary antibodies (SA00001-1; SA00001-2, Proteintech, dilution WB: 1:5000). Membranes were then washed thrice with PBST, and the enhanced chemiluminescence substrate (Millipore, USA) was used to develop the blots. The protein bands were imaged in a gel image processing system. GAPDH was used as the protein loading control. Relative expression of the target protein was determined by the corresponding ratio of the optical density values of the internal reference and the target protein band using ImageJ.

### Immunofluorescence (IF)

The neurons were fixed with 4% paraformaldehyde for 5 min and then washed thrice with PBS for immunofluorescence. The neurons were permeabilized with 0.3% Triton X-100 in PBS for 5 min and then blocked with 5% goat serum for 30 min. The neurons were blocked in blocking solution and incubated with anti-MAP 2 (Ab32454, Abcam, dilution: 1:500) overnight at 4 °C. Later, the neurons were washed in PBS and stained in the dark for 30 min with a secondary antibody (Alexa Fluor488, ab150061, 1:500). After staining, neurons were washed and Hoechst stained for 5 min for nuclei staining. The labeled neurons were observed under a fluorescence microscope Imager A2 (Carl Zeiss).

### Quantitation of neurite outgrowth in vitro

Collected images were counted from at least five random fields for each well, and the five random zones were selected at four corners and one field in the center. Images were taken from at least three coverslips from different wells. Assessment of neurite outgrowth was performed by counting 30 cells per group. We designed three-technique replicates per group, and at least three biological replicates were performed per set of experiments. Single neurons were analyzed using ImageJ software with the NeuronJ plug-in, with minimal or no overlapping neurite arbors with adjacent neurons. Distances from the soma perimeter to neurite tips were measured by tracing arbors. Various parameters were evaluated for each PFC neuron, including total neurite length, the number of primary neurites, and length of the longest neurite. A primary neurite was defined as a neurite that directly emerges from the cell body. Neurite branching was measured using the Sholl analysis plug-in of ImageJ. All samples were set to a polynomial fit for a best fitting degree.

### Immunohistochemistry (IHC)

After their last behavioral test, the rats were deep anesthetized by 2% pentobarbital perfusion through the left ventricle using saline, followed by 4% paraformaldehyde. The removed brains were postfixed overnight in the same fixative and cryoprotection until they sank to the bottom in 30% sucrose at 4 °C. The serial 25 μm brain coronal sections cut using a cryostat microtome (Leica, Wetzlar, Germany) were mounted on gelatin-coated slides. For immunohistochemical staining, brain sections were pretreated with 0.3% hydrogen peroxide for 30 min in methanol, washed in phosphate-buffered saline (PBS), and incubated in 0.3% Triton X-100 for 10 min at 4 °C. The sections were blocked in goat serum for 1 h 22 °C and washed with PBS. Later, in the blocking solution at 4 °C, the sections were incubated for 48 h with a primary antibody NRXN1 (GTX54845, GeneTex, dilution, 1:30). The sections were again incubated in secondary antibody anti-rabbit at 37 °C for 30 min after rinsing in PBS. Sections were rinsed in PBS and visualized using the DAB (Maxvision Technology, Futian, China) as the chromogen. Other than the omission of the primary antibodies, the negative controls were prepared using the same procedure.

#### TMT labeling and MS-based quantitative proteomics

The MS/MS spectra data was searched against a protein sequence databases, including all entries from the Rat UniProt database (Swiss-Prot 16,768 and TrEMBL 62,460 total of 79,228 protein forms, 2015). The searches were conducted by applying a 20-ppm precursor ion tolerance and requiring each peptide N-/C termini to allow a maximum of two missed cleavages to adhere to the trypsin protease specificity. The experimental throughput for protein quantitation was enhanced using the six-plex labeling scheme allowing up to six samples to be compared in a single MS run. The six samples containing 30 μg of peptides each were labeled in duplicate using TMT-129 and 130 for the KD group and TMT-126, 127, and 128 for the Nc group. With methionine oxidation being set as the variable modification, peptide N termini and lysine residues were set as the static modifications. By applying the target-decoy database search, a false discovery rate (FDR) of 1% on the protein level was achieved with the assignment of the MS2 spectra. A percolator (64-bit version) was used for filtering. The intensity of the signal closest to the theoretical m/z value and a 0.02 m/z window centered on the theoretical m/z value of each of the six reporter ions were recorded for qualification. All intensity values were adjusted considering the potentially uneven TMT labeling and the sample handling variance for each labeled channel. The total signal intensity across all peptides quantified was summed for each TMT. Applying the Friedman test with a Benjamini-Hochberg multiple test correction with *P* < 0.00134 cutoffs in Scaffold 4.5 (Proteome Software), the statistical significance of the changes in protein enrichment was determined by analyzing the variance of the individual peptides across samples.

#### Automated capillary Western dot blot analysis

Following the manufacturer’s guidelines, the samples were prepared and analyzed (ProteinSimple, San Jose, CA, USA). Four sample volumes were denatured at 95 °C for 5 min after mixing with one fluorescent 5 × master mix volume containing 200 mM dithiothreitol (ProteinSimple). Primary antibodies against ANXA4 (Ab256456, Abcam, dilution: 1:20), GRB2 (36344s, CST, dilution1:50), ANXA1 (32943t, CST, dilution: 1:50), and GAPDH (10494, Proteintech, dilution 1:100) were diluted in antibody diluent (ProteinSimple). The primary and secondary antibodies, the biotinylated ladder, the chemiluminescent substrate, the separation matrix (12–230 kDa), the stacking matrix, and the prepared samples were transferred into an array plate of 384 wells. After adding the Simple Western assay buffers into the system tray and the insertion of capillaries, the prepared assay plate was placed into the Simple Western machine (Sally Sue; ProteinSimple). A total of 40 nL was the injection volume of each sample. The machine automatically carried out all subsequent separation, immunodetection, and analysis steps. To calculate the area under the curve (AUC) of each peak, analyze the signal peaks automatically, and visualize the Simple Western lanes, the Compass software (Atlanta, GA, USA) was used.

#### Statistical analysis

Data were analyzed with GraphPad Prism 7 and were used to analyze the data and calculate the mean value ± standard deviation (SD). All experiments were repeated at least three times. The identification of the normal distribution was performed using the Kolmogorov-Smirnov test, and the Levene’s test was used to check the homogeneity of variance. Unpaired *t*-tests were used for data analysis between groups. One-way analysis of variance (ANOVA) was used for multiple data analysis. Tukey’s post hoc test was performed to obtain *P*-values and adequately assess differences to determine significance following one-way ANOVA; group main effect, *P* < 0.05, was considered to indicate a statistically significant difference.

## Results

### The efficiency of NRXN1 knockdown in vitro and in vivo

**Fig. 1:**
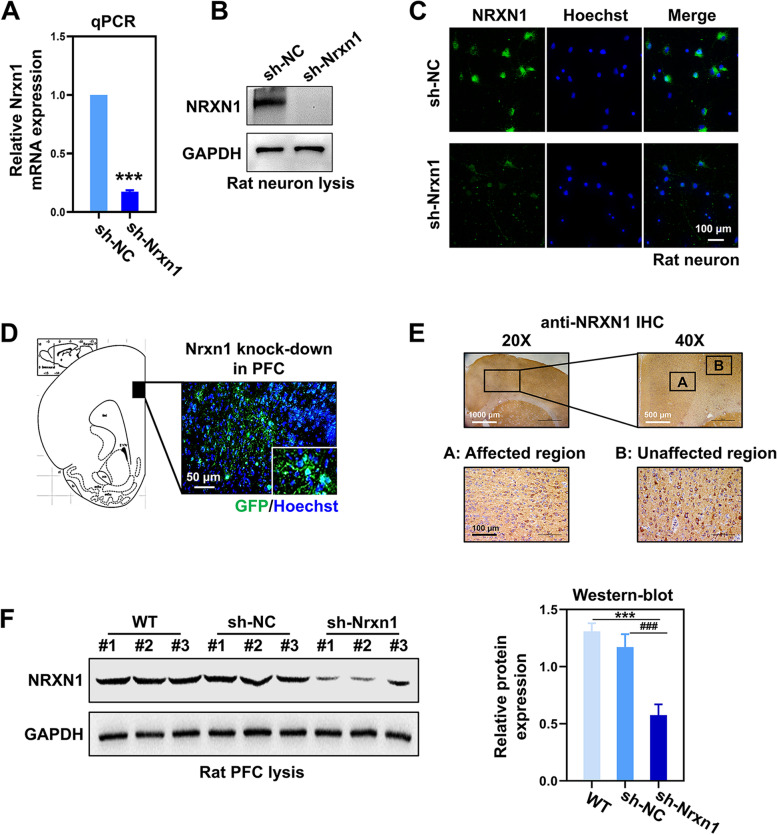
The efficiency of NRXN1 knockdown in vitro and in vivo. **A** The mRNA level of NRXN1 in primary neurons between Sh-Nc group and Sh-NRXN1 group, *P* < 0.001, *t*-test. **B** The protein level of NRXN1 in primary neurons. **C** Representative immune-stained images of NRXN1 in rat neurons. Scale bar, 50μm. **D** The image of GFP-reporter virus vectors in the prelimbic region of the PFC in rats. **E** The protein expression of NRXN1 was detected by immunohistochemistry (IHC) in brain sections of rats; representative images of the virus-affected region and unaffected region are displayed, respectively. **F** The protein level of NRXN1 in SD rats. *F* (2, 6) = 51.59, *P* < 0.001, one-way ANOVA. These data were presented as mean ± SD of three independent experiments. *P < 0.05, **P < 0.01, ***P < 0.001 versus WT group. #*P* < 0.05, ##*P* < 0.01, ###*P* < 0.001 versus Sh-Nc group

The mRNA and protein levels of NRXN1 were evaluated to investigate the knockdown efficiency of NRXN1. After being transfected for 48 h, the mRNA and protein levels of NRXN1 were effectively suppressed in primary neurons (Fig. 1 A and B). Immunofluorescence staining showed that the expression of NRXN1 was remarkably decreased (Fig. 1C). The NRXN1-KD rat model was established via stereotactic microinjection of adeno-associated virus vectors targeting NRXN1. The diffusion range of the GFP-reporter virus vectors was detected in PFC (Fig. 1D). The NRXN1 downregulated expression was confirmed by immunohistochemistry (IHC) and protein levels in rat’s brain sections (Fig. 1 E and F and Fig. S1A and S2A). These results indicated that NRXN1-KD models were successfully constructed in vitro and in vivo.

### Anxiety-like behavior and abnormal social behavior of Sh-NRXN1 rats

**Fig. 2:**
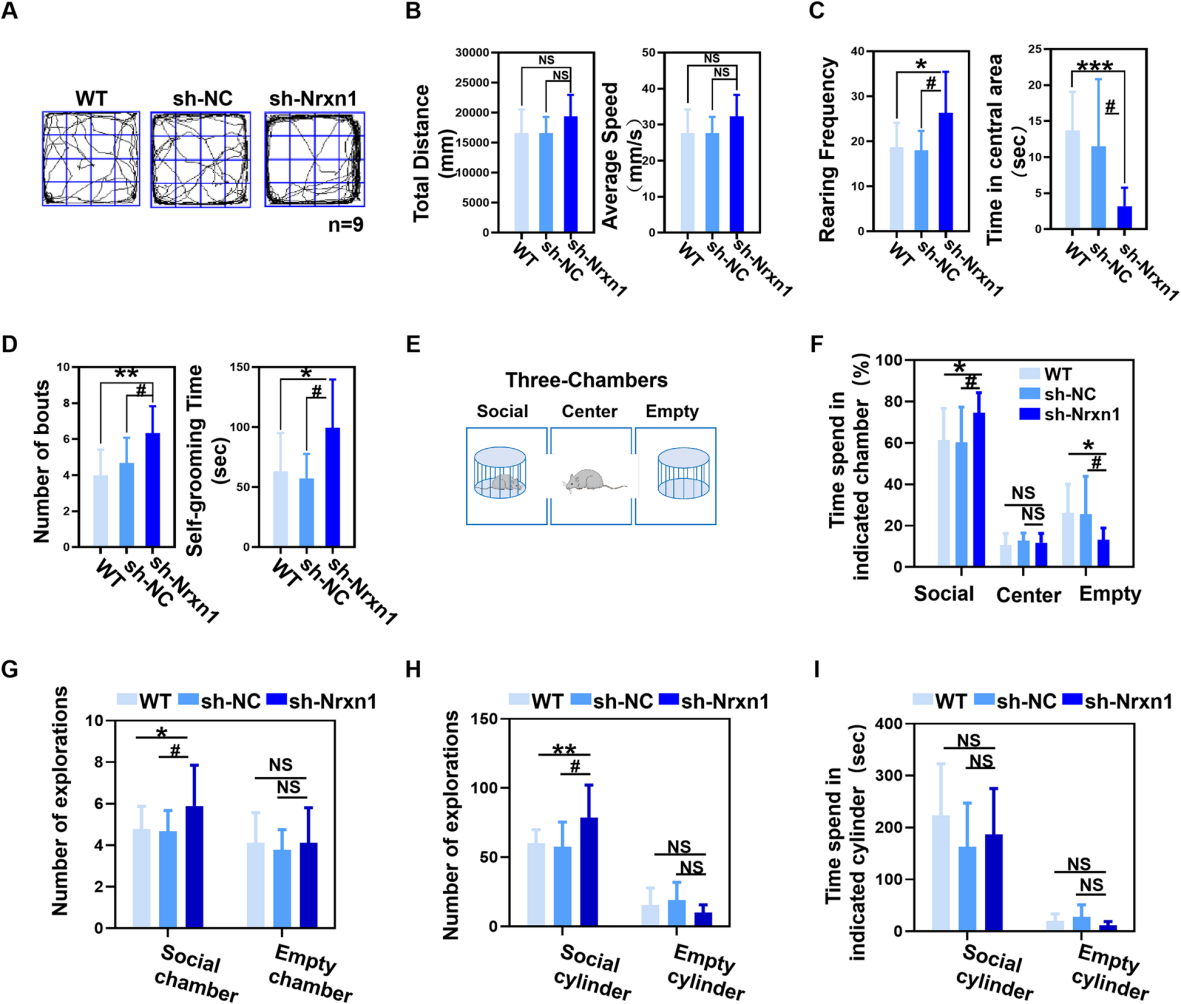
Sh-NRXN1 rats were manifested aberrant locomotive activity and social behaviors. **A** Representative traces from WT, Sh-Nc, and Sh-NRXN1 rats in open-field test. Wild type (*WT* = 9), Sh-Nc (*n* = 9), Sh-NRXN1 (*n* = 9). **B** Total distance travelled *F* (2, 24) = 2.46, *P* > 0.05 and average speed *F* (2, 24) = 1.91, *P* > 0.05 in open-field test. **C** Rearing frequency *F* (2, 24) = 4.42, *P* = 0.02 and time in central area F (2, 24) = 8.23, *P* = 0.002 obtained from open-field test. **D** Number of bouts *F* (2, 24) = 4.58, *P* = 0.02, and self-grooming time *F* (2, 24) = 4.61, *P* = 0.02, in open-field test. **E** Schematic representation of the three-chamber social interest task. Wild type (*WT* = 9), Sh-Nc (*n* = 9), Sh-NRXN1 (*n* = 9). **F** Percentage of time spent in the chamber containing the social object *F* (2, 24) = 3.5, *P* = 0.048, the center chamber F (2, 24) = 1.058, *P* > 0.05, or the empty chamber F (2, 24) = 3.49, *P* = 0.048. **G** The number of explorations between social chamber *F* (2, 24) = 4.16, *P* = 0.028, and empty chamber *F* (2, 24) = 0.275, *P* = 0.76. **H** The number of explorations between social cylinder *F* (2, 24) = 5.2, *P* = 0.013, and empty cylinder *F* (2, 24) = 1.49, *P* = 0.245. **I** Time spent on social cylinder *F* (2, 24) = 1.01, *P* = 0.36, and empty cylinder, respectively. These data were presented as mean ± SD of three independent experiments. NS > 0.05, *P < 0.05, **P < 0.01, and ***P < 0.001 versus WT group. #*P* < 0.05, ##*P* < 0.01, ###*P* < 0.001 versus Sh-Nc group

NRXN1 dysfunction is closely related to a variety of neurological disorders. In addition to the role in executive function, the PFC plays an essential role in social cognition and emotional regulation. There is evidence that the effects of prefrontal metabolic levels can lead to abnormal behavior. Thus, the behavior of Sh-NRXN1 rats was measured by an open-field test (OFT) (Fig. 2A and Fig. S1B) and a three-chamber social interaction test (Fig. 2E). There were no statistical differences among the three groups in terms of total distance and average speed (Fig. 2B). Nevertheless, Sh-NRXN1 rats showed an increase in rearing frequency and spent less time in the center of the field than Sh-Nc and WT rats (Fig. 2C), suggesting that Sh-NRXN1 rats were more indicative of increased exploration or possibly repetitive behavior. Furthermore, the number of bouts and self-grooming time was significantly upregulated in Sh-NRXN1 rats (Fig. 2D). These results indicated that NRXN1 deficiency might increase automatic and repetitive phenotype behaviors. Sh-NRXN1 rats spent significantly more time interacting with the strange rats than other groups (Fig. 2F), suggesting that the Sh-NRXN1 rats displayed an increased “social interaction” behavior. To better understand the social interaction process of Sh-NRXN1 rats, we found that downregulation of NRXN1 increases the entries into the social chamber (Fig. 2G) and the number of contacts with the social cylinder (Fig. 2H). However, the time of interacting with the social cylinder was not prolonged (Fig. 2I). This suggests that this more active social pattern is like a reciprocating movement because the animals do not interact for longer. Our results demonstrate the inhibitory role of NRXN1 in PFC-induced neurodevelopmental behaviors in SD rats, including anxiety-like behaviors, repetitive behavioral phenotypes, and social abnormalities.

### The effects of NRXN1 downregulation on neurite outgrowth in primary rat neurons

**Fig. 3:**
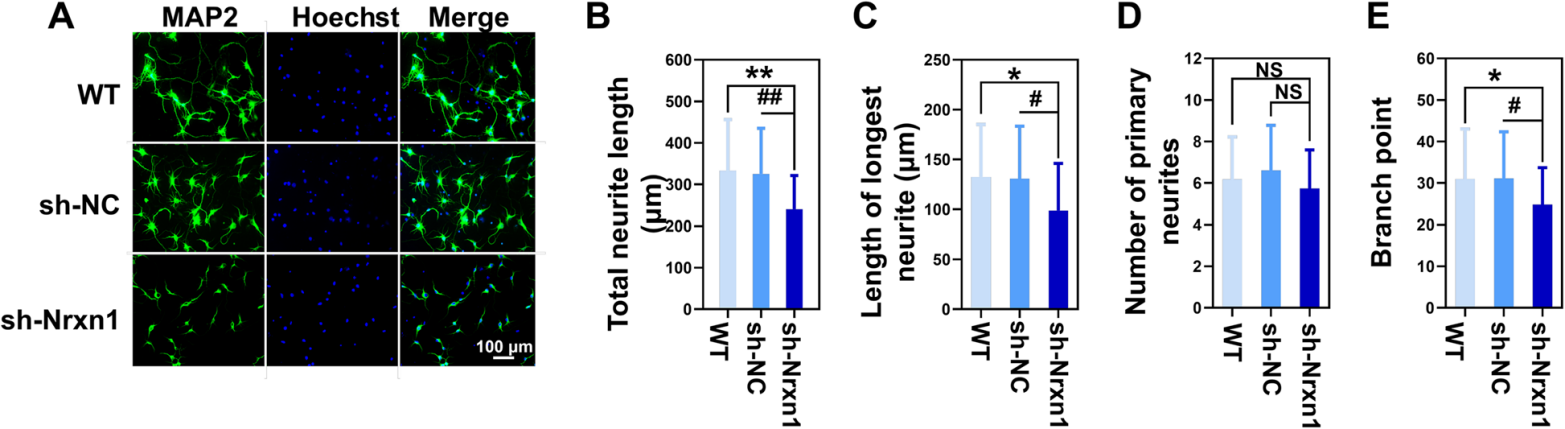
The effects of NRXN1 downregulation on neurite outgrowth in primary rat neurons. **A** Representative immune-stained images for microtubule association protein-2 (MAP 2) in primary cultured cortical neurons were shown. Scale bar, 50 μm. Wild type (WT), Sh-Nc, Sh-NRXN1. **B** Quantification of total neurite *F* (2, 87) = 7.05, *P* = 0.001. **C** Quantification of the length of longest neurite *F* (2, 87) = 4.12, *P* = 0.019. **D** Statistics on the number of primary neurites *F* (2, 87) = 1.36, *P* = 0.261. **E** The number of branch points *F* (2, 87) = 3.29, *P* = 0.042. The neurite length was determined by ImageJ. These data were presented as mean ± SD of three independent experiments. NS > 0.05, *P < 0.05, **P < 0.01 versus WT group. #*P* < 0.05, ##*P* < 0.01 versus Sh-Nc group

To investigate the effects of NRXN1 on neurite growth, MAP 2 was used to visualize the length and distribution of neurites by immunofluorescence (IF) staining in primary rat neurons. MAP 2 staining showed the morphology of primary cultured neurons transduced with lentiviral vectors (Fig. 3A and Fig. S1C). Knockdown of NRXN1 significantly reduced the length and branch points of neurites. Quantitative analysis revealed that NRXN1-KD neurons exhibited a significant decrease in length of longest neurite and total neurites compared with the neurons transfected with the WT and Sh-Nc group (Fig. 3 B and C). There was no statistical difference among the number of primary neurites in the three groups (Fig. 3D). However, the number of neuron branch points in the Sh-NRXN1 group was significantly lower than in the other two groups (Fig. 3E), indicating that NRXN1 may affect the complexity of neural networks. Our results revealed that NRXN1 was important in neurite formation and outgrowth in primary rat neurons.

### Proteomic analysis of rat PFC neurons from Sh-NRXN1 and control group

**Fig. 4:**
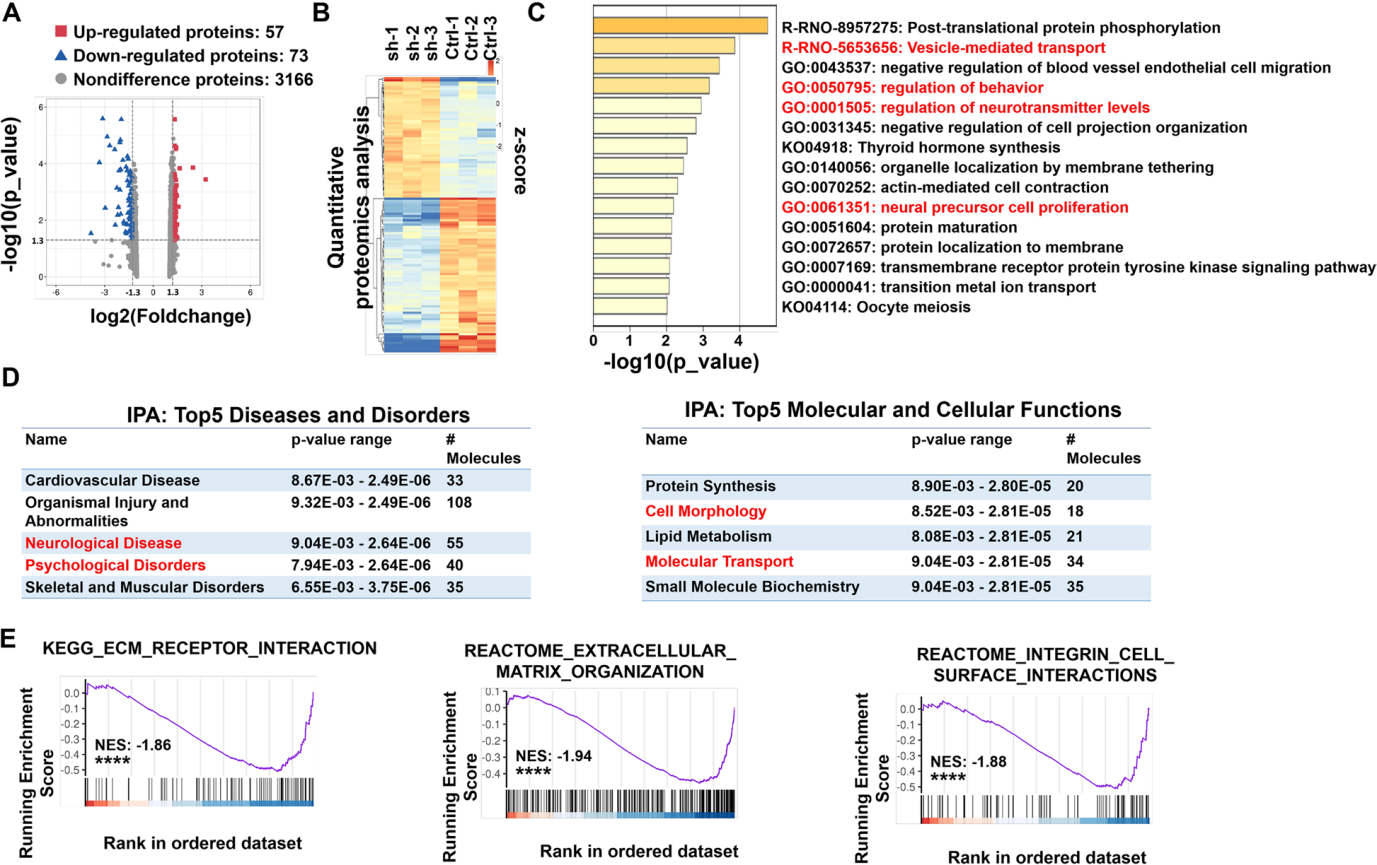
Functional enrichment analysis of proteomic results. **A** Volcano plot of all 3296 identified proteins quantified by proteomic analysis. **B** Heatmap was plotted based on the levels of the differentially expressed proteins. The color intensity indicates the expressional protein level as displayed: red, highest; green, lowest. **C** Biological pathway analysis of differently expressed proteins was performed in Metascape. The top 15 significantly enriched terms were displayed. Color-coded *P*-values indicate the significance of the enrichment for each bin as indicated. Our experiment observed abnormal behaviors and impairment in neurite outgrowth in Sh-NRXN1 rats. Combined with the reported correlation of NRXN1 on neurotransmitter release, the features we focus on are highlighted in red. **D** The proteomic result was uploaded into ingenuity pathway analysis (IPA). Top-ranked significantly enriched disease and disorder (left) and molecular and cellular (right) pathways in the IPA reference database were listed. **E** The importance of the downregulated protein-coding gene was assessed using a GSEA approach. The position of subjected genes in the ranked list was represented by black bars, and the purple curve represents the running enrichment score (ES). The normalized enrichment score (NES) is the primary statistic for assessing gene set enrichment results. GSEA revealed a significant enrichment of ECM_RECEPTOR_INTERACTION (left), EXTRACELLULAR_M-ATRIX_ORGANIZATION (middle), and INTEGRIN_CELL_SURFACE_INTERA-CTIONS (right), ****P < 0.0001

The proteomic analysis was evaluated because the core effect for NRXN1 to work is protein-protein interaction. There were 130 proteins with significantly different expressions compared to the control group (Fig. 4A and Table S2), including 57 upregulated proteins (TOP 5 are Usp13, Setdb1, Hist1h1a, Spock3, and Ror2) and 73 downregulated proteins (TOP 5 are Dpp9, Rlf, Grk3, NRXN1, and Epas1). The different expression proteins were evaluated by hierarchical clustering analysis and were plotted as a heatmap (Fig. 4B). To gain insight into the biological implication of these altered proteins, all significantly changed proteins were analyzed by functional enrichment based on the GO/KEGG terms, canonical pathways, and hallmark gene sets. Top-ranked representative-enriched pathways are shown in Fig. 4C. Pathways highlighted in red were closely related to the physiological function of neurons. Ingenuity pathway analysis (IPA) was performed using all significantly changed proteins in Sh-NRXN1 group. The top five terms identified by IPA were found in two categories, including “molecular and cell functions” and “disease and disorders” (Fig. 4D). Importantly, terms that are strongly associated with mental diseases were revealed by IPA (marked in red). Suppression of NRXN1 expression may lead to the degradation of binding partners. Thus, by using gene set enrichment analysis (GSEA), we further analyzed the function and pathway influenced by proteins downregulated with NRXN1. Significantly impaired pathways concerned the extracellular matrix, cell membrane, and morphologic change (Fig. 4E).

### Proteins related to cell morphology and membrane structure were downregulated in NRXN1-KD rats

**Fig. 5:**
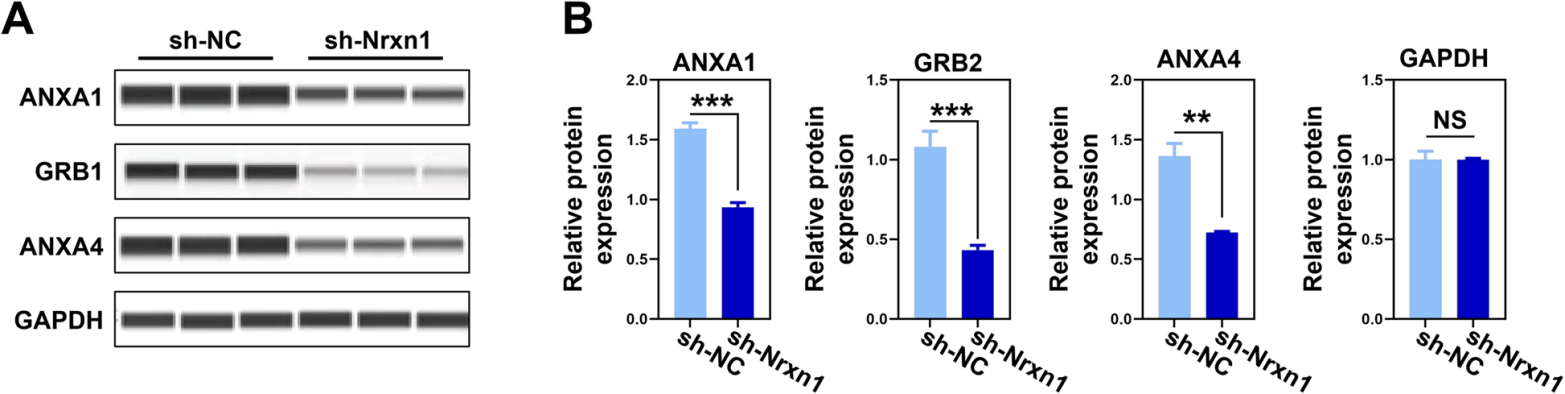
Immunoblotting validation of key proteins centering on cell membrane structure. **A** Automated capillary Western blot validation of ANXA1, GRB2, and ANXA4. **B** The expression of ANXA1 (*t* = 14.216, *df* = 4, *P* < 0.001), GRB2 (*t* = 10.805, *df* = 4, *P* < 0.001), and ANXA4 (*t* = 10.118, *df* = 4, *P* = 0.009) was quantified according to the corresponding bands. GAPDH was used as the internal reference marker and loading control for normalization. These data were presented as mean ± SD of three independent experiments. *NS* > 0.05, **P < 0.01 and ***P < 0.001, compared with the corresponding control group

To further determine the expressions of proteins identified by TMT-based quantitative proteomics, three differentially expressed proteins related to cell morphology and membrane structure were chosen, including Annexin 1 (ANXA1), Annexin 4 (ANXA4), and growth factor receptor-bound protein 2 (GRB2). Consistent with the proteomic data, we found that the expression of ANXA1, ANXA4, and GRB2 was significantly decreased following the knockdown of NRXN1 in prefrontal neurons (Fig. 5 and Fig. S2C).

## Discussion

Neurodevelopmental disorders (NDDs) include syndromes characterized by abnormal central nervous system development, affecting learning, cognition, emotion, and memory [1]. Environmental and genetic factors contribute to neuronal impairment, resulting in NDDs [30, 31]. NDDs are heterogeneous and may manifest in the form of comorbidity. Patients with attention deficits may show autistic features. Likewise, approximately half of individuals with ASD have ADHD [32, 33]. This implies that NDD syndromes may partially share overlapping genetic abnormalities. Therefore, elucidation of the shared pathogenic mechanisms of NDDs will help to explore effective diagnosis and treatment methods.

NRXNs, predominantly NRXN1, are related to SCZ, ASD, and ADHD. Thus, NRXN1 has emerged as a significant risk gene for NDDs. The significant disease association with NRXN1 has encouraged efforts to clarify mechanistic insights into how this gene influences neuronal behaviors, including hypotonia, language delays, and intellectual disability; neurexin-1deletions predispose to a broad spectrum of developmental disorders besides autism and SCZ [34]. In a case of a 7-year-old boy with autism, deletions of the NRXN promoter and exons 1–5 were identified, the first evidence linking neuropsychiatric disorders to NRXN1 deletions [35]. Moreover, other studies showed compound heterozygous NRXN1 deletions in patients with epilepsy, early developmental delay, and intellectual disability [36–38]. PFC plays a significant role in executive functions, including cognitive flexibility and attention. The NRXN1α deletion altered the PFC regions’ metabolism, as suggested by the available evidence [21]. Besides, the heterozygosity of *α*-neurexin-1 also reduced the efficiency of the functional brain networks. The PFC may be dysfunctional in humans from the clinical cases reported with 2p16.3 deletions. In a cohort of 236 controls and 260 ASD patients, two deletions were identified in the ASD patients in the initial exons of NRXN1 [39]. Proposing the possibility of a gene-dose effect, a slight reduction in the NRXN1 protein was sufficient to generate an ASD phenotype [40]. The upstream of exon 1 contained the promoter of the *a*-neurexin transcripts. In contrast, the neurexin-1β promoter is in the intron downstream of exon 17. Therefore, transcripts from the affected homolog could be expected from the exon 1 deletion, a complete absence of the *α*-neurexin transcript but not NRXN1β [40–42]. In human diseases, the copy number mutations are usually duplications of high dosage-sensitive genes and hemizygous deletions and knockouts in rare instances [18]. In contrast, complete knockouts are usually observed in rodents. In our prefrontal neurons culture, interfering viruses were designed to target exon 1, a site where copy number variation has been repeatedly reported in clinical cases. Our viral interference efficiency was close to 80% in vitro and 50% in vivo. Virus infection efficiency in vitro was significantly higher than in vivo, possibly due to virus diffusion in the brain tissue. Therefore, our model more realistically simulates clinical patients with NRXN1 mutation, and our experimental results are pretty representative.

We established the Sh-NRXN1 rat model via stereotactic microinjection of an adeno-associated virus targeting NRXN1 in the prefrontal lobe [43]. PFC regions serve essential cognitive functions related to behavior’s social, emotional, and motivational aspects [20]. Defection in PFC function may lead to abnormal behavior. Although behaviors are various, they all suggest that NDDs may affect cognitive levels [44]. Executive functions are the top-down processes required to change or override automatic responses and are affected by many factors [45]. We used Morris water maze test to evaluate the learning and memory phenotypes of Sh-NRXN1 rats in our previous study [29]. Disorganized PFC structure [46], altered PFC thickness [47], changes in PFC connectivity [48], and dendritic integration [49] are often observed in individuals affected by NDDs. Such anatomical changes in PFC are thought to underlie impaired social and repetitive phenotypes [50, 51]. In this study, the open-field test was used to evaluate the locomotor activity and anxiety behavior, while the three-chamber sociability test evaluated the social behavior of the rat. Compared to control rats, Sh-NRXN1 rats spent significantly less time near the center of the open-field test. They used more time near the periphery, indicating increased anxiety-like behaviors. Rearing frequency significantly increased in the Sh-NRXN1 group, and increased vertical movement suggested exploratory elevation in Sh-NRXN1 rats. Grooming events dramatically increased in the Sh-NRXN1 group, indicating increased repetitive behaviors. In a three-chamber test, all rats spent more time in the social chamber compared to the empty chamber. Interestingly, in the exploration of the social chamber, the exploration time and frequency of the Sh-NRXN1 rats were significantly increased. The frequency of interaction with the social cylinder of Sh-NRXN1 rats increased significantly, but the duration of interaction did not extend, suggesting that Sh-NRXN1 rats displayed more frequent social activities.

In this study, behavioral abnormalities, including anxiety-like behavior, repetitive behavior, and abnormal social phenotype were observed in PFC-specific Sh-NRXN1 SD rats. Our findings are generally consistent with previous studies [15–17] and reconfirm the association of NRXN1 with abnormal behavior in NDDs. The NRXN1 mutations have been repeatedly reported in patients with ASD [1–3, 6], and the core symptoms of ASD are social deficits with or without repetitive behaviors. Our experiment verified that NRXN1 was related to repetitive behavior, which was consistent with the results reported by Etherton team [14]. Interestingly, the rats in our study did not exhibit social deficits (e.g., more extended social interaction with inanimate objects, less sniffing time, or duration time with the social animal). Conversely, the rats presented more positive social activities. In addition to abnormal social patterns, the Sh-NRXN1 rats also revealed significantly increased repetitive and exploratory behavior. Therefore, we could hypothesize that NRXN1 downregulation impairs rats’ learning and memory ability and thus impact cognition [18]. With the influence of NRXN1 knockdown on social memory [17], Sh-NRXN1 rats may spend more time or explore more frequently to invest social cues given off, which is consistent with the appearance of NDD children with mental impairments who improve their cognitive and operational performance through repetition. Our experiment also shows significant anxiety-related behavior in PFC-specific Sh-NRXN1 rats, which is consistent with Grayton’s study [16]. This might be related to the critical role of PFC in social cognition and emotion regulation. Given the strong correlation of repetitive behavior with autism, this “active” social pattern with high back-and-forth movement frequency could also be stereotyped with no actual social content. This behavior becomes starker in the presence of anxiety. However, Etherton and colleagues investigated no effects of *α*-neurexin-1 HET deletion in social behavior tests [15]. There is also disagreement about the influence of anxious behaviors, although the Sh-NRXN1 group in our experiment showed a significant tendency toward anxiety [15–17]. There may be multiple reasons for the difference in the results. Studies conducted on SD rats differed from the earlier experiments in mouse species used in that they had a single genetic background rather than a mixed genetic background (C57BL6/SV129). Gene expression levels also need to be considered. Homozygous or heterozygous gene knockout mice were used in previous experiments. In contrast, PFC-specific Sh-NRXN1 rats were used in our experiment, considering the special role of the prefrontal lobe in neurodevelopmental disorders. The NDDs experimental subjects should be juvenile, while previous studies only used adult mice. The observed differences might be because many impairments at young age become less evident in adulthood as other abilities compensate for them. These unduplicated phenotypes provide richer insights into a specific prefrontal knockout in adolescence.

Abnormal neural network connections and neuronal damage are the basis of attention deficit [52–54]. Previous studies of heterozygous deletion of the α-neurexin 1 have shown alterations in select cortical regions and the cerebral metabolism in the neural system. Through the reduced thalamic “rich club” regional interconnectivity and the thalamic PFC, the *α*-neurexin-1 heterozygosity was found to reduce the efficiency of brain functional networks through the last thalamic “rich club” of PFC [21]. Previous studies focused on the effects of NRXN1 on synaptic composition and function [23, 29]. However, more studies are required to fully understand the effect of NRXN1 on neurites. Although there are contradictory views about the NRXN1 influence on neurite outgrowth, we hypothesized that NRXN1 might affect neurite outgrowth, thereby influencing behavior. Using primary neurons obtained from the rat PFC, we observed that NRXN1 downregulation impaired neurite outgrowth. Neurites are essential for proper neuronal function, as the shape of the dendritic arbor determines the receptive field of a neuron and axon growth defines the extent of neuronal output. On the other hand, synapses are the terminal structures of neural processes, and the impact of neural processes will inevitably lead to synaptic damage. Thus, generating and maintaining proper neurite arborization is critical for normal neural circuit function [55]. The impairment of the delicate balance between neurite outgrowth and retraction may induce NDDs. Therefore, we further clarified the potential mechanism of NRXN1 in neurite outgrowth, especially at the protein level.

Our TMT-based proteomic analysis identified 130 differently expressed proteins as closely related to neurons’ physiological function, mental diseases, extracellular matrix, cell membrane, and morphologic change. Neurons need to form correct connections to orchestrate a functional neuronal circuitry during brain development. An intact cell membrane structure is essential for correct neuronal function as improper neuronal connectivity results in NDDs. Previous studies have reported that ANXA1 has neuroprotective effects on the central nervous system [56]. GRB2 is a scaffolding adaptor protein that is crucial in transmitting signals that control cell growth and differentiation [57]. The destruction will induce the degradation of interacting proteins. Thus, downregulated proteins may be the potent partner of NRXN1. Given NRXNs’ central role in neuronal physiology, the specific mechanism needs to be clarified.

## Conclusions

In conclusion, NRXN1 depletion in the medial PFC induces anxiety-like behavior, and abnormal social phenotypes in our observations are consistent with previous studies [16, 17]. In rats, downregulation of NRXN1 was associated with neurite growth. Our proteomics analysis revealed the impairment of NRXN1 protein-induced disorder in neuron proteome, especially in the cell membrane. ANXA1, ANXA4, and GRB2 were chosen to verify the results of TMT-based quantitative proteomics-influenced proteins. This disorder in the neuron proteome may be the underlying mechanism for abnormal behavior in NDDs. Many proteins have been identified as NRXN1binding partners, and it could be hypothesized that NRXN1 impairment will damage the protein-protein interaction, leading to neurodevelopmental disorders. However, more research is needed to reveal these changes’ molecular mechanisms. Our results confirm the association of NRXN1 with abnormal behavior in NDDs and provide richer insights into NDDs’ prefrontal knockdown in adolescence. Our findings potentially expand the NRXN1 interactome and are helpful for human health.

## Supplementary Information

**Additional file 1: Figure S1.** (A) The protein expression of NRXN1 was detected by immunohistochemistry (IHC) in brain sections of rats among WT, Sh-Nc, Sh-NRXN1 groups. NRXN1 expression in the PFC injection region was decreased of Sh-NRXN1group (Related to Figures 1E). (B) The distance traveled in central area and peripheral in OFT test. Sh-NRXN1 rats traveled less distance in central area and showed an increase distance in peripheral area than Sh-Nc and WT rats, suggesting that Sh-NRXN1 rats were more anxious (related to Figures 2A). We found that down regulation of Nrxn1 decreases the number of transition from the peripheral area to the central area, it was the underlying anxiety phenotype rather than the motor phenotype. (C) Representative images of primary PFC neurons among WT, Sh-Nc, Sh-NRXN1 groups (related to Figures 3A). Using Sholl analysis to characterize the morphological characteristics of the primary PFC neurons (related to Figures 3E), we found that down regulation of NRXN1 in primary PFC neurons exhibited a significant decrease in the number of neurite intersections between 50 μm from the cell body compared to the Sh-Nc group and WT group F (2, 87) =5.08, *P*=0.008, one-way ANOVA, Tukey's post hoc. These data were presented as mean ± S.D. of three independent experiments. NS > 0.05, * *P* < 0.05 versus WT group. # *P* < 0.05 versus Sh-Nc group. **Figure. S2.** (A) Uncropped western blot image of anti-NRXN1 and anti-GAPDH, western blot to probe NRXN1 protein in the PFC lysates of Nrxn1-KD models. Samples were obtained from prefrontal tissue of three rats treated in the same way (Related to Figures 1F). (B) The virtual gel image of automated capillary western dot blot analysis, validation of ANXA1, GRB2 and ANXA4. (C) Electropherograms overlay traces of PFC neuron lysates from Sh-Nc and Sh-Nrxn1 group sample (Related to Figures 5B). **Table S1.** Primer sequences of *NRXN1* and *GAPDH*. **Table S2.** Differentially expressed proteins in prefrontal neurons.

## Data Availability

The data that support the findings of this study are available from the corresponding author with reasonable request.

## Abbreviations

NDD: Neurodevelopmental disorder
NRXN1: Neurexin-1
PFC: Prefrontal cortex
ASD: Autism spectrum disorder
ADHD: Attention-deficit hyperactivity disorder
NLGN3: Neuroligin-3
TMT: Tandem mass tag
ID: Intellectual disability
