# Supplementary material for: NRXN1 depletion in the medial prefrontal cortex induces anxiety-like behaviors and abnormal social phenotypes along with impaired neurite outgrowth in rat
Source: J Neurodev Disord. 2023 Feb 3;15:6. doi: 10.1186/s11689-022-09471-9 (PMC9896742; doi:10.1186/s11689-022-09471-9)
Supplement: Supplementary file 1 — Additional file 1: Figure S1. (A) The protein expression of NRXN1 was detected by immunohistochemistry (IHC) in brain sections of rats among WT, Sh-Nc, Sh-NRXN1 groups. NRXN1 expression in the PFC injection region was decreased of Sh-NRXN1group (Related to Figures 1E). (B) The distance traveled in central area and peripheral in OFT test. Sh-NRXN1 rats traveled less distance in central area and showed an increase distance in peripheral area than Sh-Nc and WT rats, suggesting that Sh-NRXN1 rats were more anxious (related to Figures 2A). We found that down regulation of Nrxn1 decreases the number of transition from the peripheral area to the central area, it was the underlying anxiety phenotype rather than the motor phenotype. (C) Representative images of primary PFC neurons among WT, Sh-Nc, Sh-NRXN1 groups (related to Figures 3A). Using Sholl analysis to characterize the morphological characteristics of the primary PFC neurons (related to Figures 3E), we found that down regulation of NRXN1 in primary PFC neurons exhibited a significant decrease in the number of neurite intersections between 50 μm from the cell body compared to the Sh-Nc group and WT group F (2, 87) =5.08, P=0.008, one-way ANOVA, Tukey's post hoc. These data were presented as mean ± S.D. of three independent experiments. NS > 0.05, * P < 0.05 versus WT group. # P < 0.05 versus Sh-Nc group. Figure. S2. (A) Uncropped western blot image of anti-NRXN1 and anti-GAPDH, western blot to probe NRXN1 protein in the PFC lysates of Nrxn1-KD models. Samples were obtained from prefrontal tissue of three rats treated in the same way (Related to Figures 1F). (B) The virtual gel image of automated capillary western dot blot analysis, validation of ANXA1, GRB2 and ANXA4. (C) Electropherograms overlay traces of PFC neuron lysates from Sh-Nc and Sh-Nrxn1 group sample (Related to Figures 5B). Table S1. Primer sequences of NRXN1 and GAPDH. Table S2. Differentially expressed proteins in prefrontal neurons. [file 11689_2022_9471_MOESM1_ESM.docx]

**NRXN1 depletion in the medial prefrontal cortex induces anxiety-like behaviors and abnormal social phenotypes along with impaired neurite outgrowth in rat**

Di Wu, Jiansheng Zhu, Lianghui You, Jingyu Wang, Sufen Zhang, Zhonghui Liu, Qu Xu, Xiaojie Yuan, Lei Yang, Wei Wang, Meiling Tong, Qin Hong, Xia Chi

**Supplementary Materials**

**Detailed description of open field test behavior**

The test apparatus consisted of a dark open box of Plexiglas (40 cm length × 45 cm height × 40 cm width). The open-field arena was divided-by black lines-into 16 squares of 10 × 10 cm. The central 4 squares were defined as the central zone, in which animals’ activity was regarded as a measure of anxiety (Prut and Belzung, 2003). Testing was done under 4-W cold light source above the center of the box. Rats were first habituated for 10 min in the test box. On the test day, each animal was then placed in the same corner of the open field arena, and its behavior was recorded for 10 min. The variables observed were: (a) total distance and average speed (b) the number of rearing behaviors (vertical activity, defined as raising both forepaws above the floor while balancing on hind limbs), and (c) the amount of the time spent in the central zone as defined by all forepaws being in the central 4 squares of the apparatus, (d) Time spent in self-grooming and numbers of bouts were manually recorded. (auto grooming was scored when the subject animal groomed any portion of its own body). (e) The distance the rats traveled in the central zone and peripheral area. (f) The number of transitions from the peripheral area to the central area (Helps distinguish between potentially active or anxious behaviors）（Figure. S1B）.

**Detailed description of social behavior**

Sociability was conducted as described (Moy et al., 2004). The experimental rats were allowed to habituate for 15 min. During the habituation period, a subject rat was placed in the middle chamber, where the sliding doors kept opening so that the rat moved to the entire arena freely. The duration of time in each of the two outside stimulus compartments was hand scored with stopwatches. Two cylinders with a diameter of 22 cm (40 cm in height) were placed in the outer compartments for the stimulus rats during habituation. Each rat went through an adaptation phase in random order. Following the habituation phase, rats were placed back into the center, the doors were closed, and a single unfamiliar male SD rat (age 6 weeks) was randomly placed in one of the two cylinders. Interacting was classified if the rat contacted any part of the cylinder or sniffed within 2 cm radius of the target. The duration of time spent in each chamber; the number of explorations between the social and empty chamber; the number of investigations between the social and empty cylinder; time spent on the social and empty cylinder was measured by Shanghai Jiliang Animal Behavior Analysis System (Shanghai, China).

**Behavioral Overview**

The rats were moved to the test room three days before the experiment to prevent the unfamiliar environment from affecting the results. All rats ranged from 6 to 7 weeks of age during behavioral testing. All rats were provided with ad lib food and water, and 3 were group-housed in a cage. Rats were kept on a 12 hr light/dark cycle with lights on at 7 am. We conducted the behavioral testing during the light cycle. All behavioral testing was conducted by experimenters blind to the group. More stressful behaviors were tested after less stressful behaviors.

**Supplementary Figures and Figure Legends**


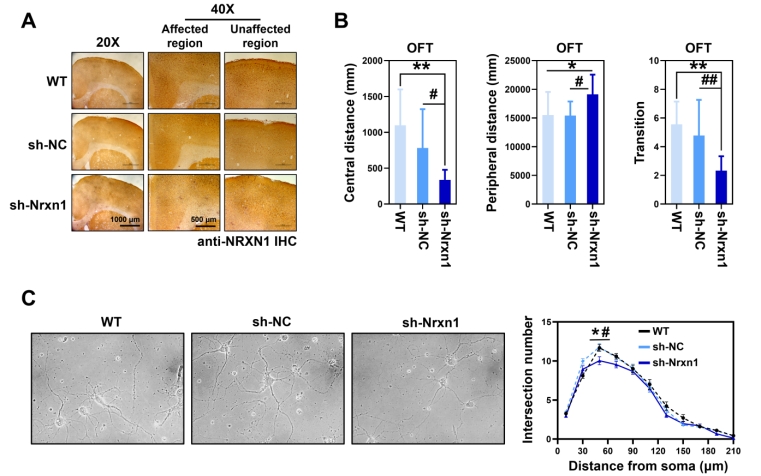


**Figure. S1**

**(A)** The protein expression of NRXN1 was detected by immunohistochemistry (IHC) in brain sections of rats among WT, Sh-Nc, Sh-NRXN1 groups. NRXN1 expression in the PFC injection region was decreased of Sh-NRXN1group **(Related to Figures 1E)**. **(B)** The distance traveled in central area and peripheral in OFT test. Sh-NRXN1 rats traveled less distance in central area and showed an increase distance in peripheral area than Sh-Nc and WT rats, suggesting that Sh-NRXN1 rats were more anxious **(related to Figures 2A).** We found that down regulation of Nrxn1 decreases the number of transition from the peripheral area to the central area, it was the underlying anxiety phenotype rather than the motor phenotype. **(C)** Representative images of primary PFC neurons among WT, Sh-Nc, Sh-NRXN1 groups **(related to Figures 3A)**. Using Sholl analysis to characterize the morphological characteristics of the primary PFC neurons **(related to Figures 3E)**, we found that down regulation of NRXN1 in primary PFC neurons exhibited a significant decrease in the number of neurite intersections between 50 µm from the cell body compared to the Sh-Nc group and WT group F (2, 87) =5.08, *P*=0.008, one-way ANOVA, Tukey's post hoc. These data were presented as mean ± S.D. of three independent experiments. NS > 0.05, * *P* < 0.05 versus WT group. # *P* < 0.05 versus Sh-Nc group.


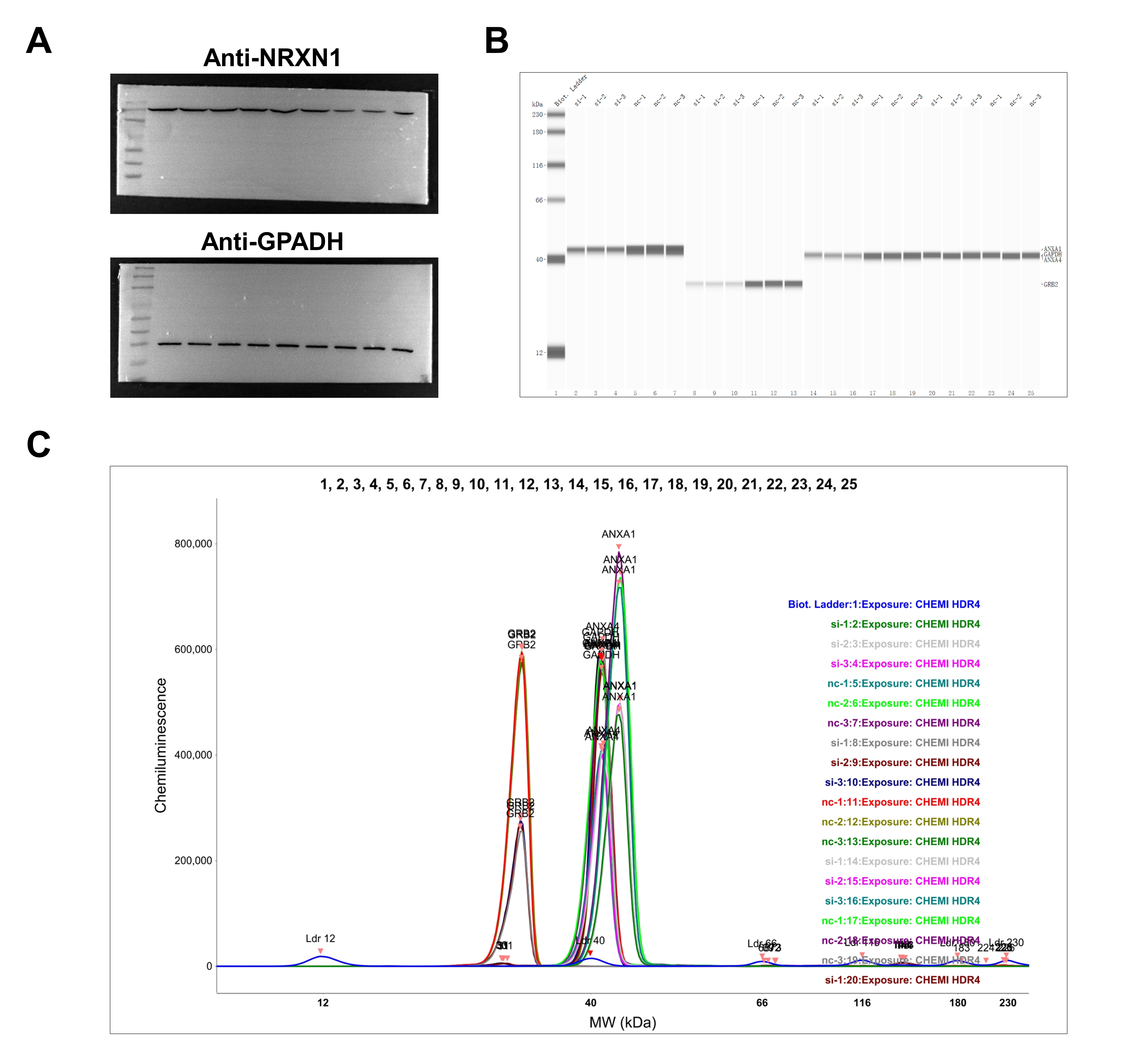


**Figure. S2**

**(A)** Uncropped western blot image of anti-NRXN1 and anti-GAPDH, western blot to probe NRXN1 protein in the PFC lysates of Nrxn1-KD models. Samples were obtained from prefrontal tissue of three rats treated in the same way **(Related to Figures 1F). (B)** The virtual gel image of automated capillary western dot blot analysis, validation of ANXA1, GRB2 and ANXA4**. (C)** Electropherograms overlay traces of PFC neuron lysates from Sh-Nc and Sh-Nrxn1 group sample **(Related to Figures 5B)**.

**Table S1 Primer sequences of *NRXN1* and *GAPDH***

| **Genes** | **Primer** | **Sequence (5'-3')** |
| --- | --- | --- |
| NRXN1 | Foward | AGGGCGTCAGCTCACAATCTTCAA |
|  | Reverse | TCTGCCGAGCTGGGTATGGT |
| GAPDH | Foward | TGCCACTCAGAAGACTGTGG |
|  | Reverse | TTCAGCTCTGGGATGACCTT |

**Table S2 Differentially expressed proteins in prefrontal neurons**

| **UniProt accession** | **Gene name** | **Protein description** | **Sh-Nrxn1 vs. Sh-Nc** | |
| --- | --- | --- | --- | --- |
|  |  |  | **Fold change** | ***P*-value** |
| A0A0G2JZ82 | Grk3 | Protein-serine/threonine kinase | -3.318333512 | 9.07505E-05 |
| D3ZUT1 | Spock3 | SPARC/osteonectin, cwcv and kazal-like domains proteoglycan 3 | 1.555824804 | 0.003317814 |
| A0A140TAA3 | Ralgapa1 | Ral GTPase-activating protein subunit alpha-1 | -2.099665789 | 0.000175208 |
| A0A0G2K3A8 |  | Uncharacterized protein | -2.89776671 | 0.000178218 |
| P28233 | Gja6 | Gap junction alpha-6 protein | -1.816706277 | 0.011310837 |
| M0R781 | Dpp9 | Dipeptidyl peptidase 9 | -6.889007002 | 0.000939352 |
| Q8R4A1-2 | Ero1a | Isoform 2 of ERO1-like protein alpha | -1.56174487 | 0.005556707 |
| Q9R1T3 | Ctsz | Cathepsin Z | -1.671926602 | 0.000130562 |
| A0A0G2JSH5 | Alb | Serum albumin | -2.054968031 | 1.79108E-05 |
| Q66HK8 | Cyp4f1 | Cytochrome P450 4F1 | -2.146980996 | 0.003489649 |
| P02767 | Ttr | Transthyretin | -1.586379202 | 5.52273E-05 |
| P01322 | Ins1 | Insulin-1 | -2.021038217 | 1.45413E-05 |
| Q4V8P9 | Tfap2c | Transcription factor AP-2 gamma | -1.520183658 | 0.000611627 |
| Q5M872 | Dpep2 | Dipeptidase 2 | -2.006554362 | 0.00015923 |
| D3ZX56 | Ddx55 | RNA helicase | -2.371190403 | 3.17763E-05 |
| A0A096MJN8 | Cmtm5 | CKLF-like MARVEL transmembrane domain-containing 5 | -2.838577415 | 1.11632E-05 |
| F1LQR8 |  | Uncharacterized protein | -3.278497893 | 4.71357E-05 |
| F1LRK3 | Adcy4 | Adenylate cyclase type 4 | -2.683983514 | 2.29749E-05 |
| F1MA14 | Gucy2g | Guanylate cyclase | -2.714113123 | 7.48605E-07 |
| A0A0G2QC06 | Tf | Serotransferrin | -1.871534228 | 7.48359E-05 |
| A0A0G2K645 | Wls | Protein wntless homolog | -2.191521605 | 0.006509167 |
| F1M785 | Pdzd2 | PDZ domain-containing protein 2 | -1.606054965 | 0.0001741 |
| D4A2M3 | Tmtc1 | Transmembrane and tetratricopeptide repeat-containing 1 | -1.968687923 | 2.71173E-06 |
| D4A3K5 | Hist1h1a | Histone H1.1 | 1.640100579 | 0.000145223 |
| D3ZF13 | Ndufab1 | Acyl carrier protein | -1.505154236 | 0.002104556 |
| F1M8F6 | Myh8 | Myosin-8 | -2.235860618 | 0.00071251 |
| D4A4K4 | Vps13c | Uncharacterized protein | -1.780874804 | 0.01311185 |
| Q63158 | Sp4 | RCG21090 | -1.653432497 | 0.013677921 |
| D3ZJX1 | Rlf | Rearranged L-myc fusion | -3.838085874 | 0.029019024 |
| D3ZJ81 | Fstl4 | Follistatin-like 4 | -1.59036602 | 0.000398331 |
| D4A081 | Setdb1 | Histone-lysine N-methyltransferase | 2.452344054 | 0.000137792 |
| Q7TP01 | LOC691143 | Bq135360 | -1.513711555 | 0.001696907 |
| Q64273 | Kcnj2 | Inward rectifier potassium channel 2 | -2.053471333 | 0.000842809 |
| Q6AY48 | Pcbp3 | Poly(RC) binding protein 3 | -1.702160515 | 0.025577464 |
| D3ZDI9 | Usp13 | Ubiquitinyl hydrolase 1 | 3.243818535 | 0.00036184 |
| A0A0G2JZG3 | Klrk1 | Killer cell lectin-like receptor subfamily K, member 1, isoform CRA_d | -2.002619204 | 0.006022085 |
| D4ABX6 | Mmrn2 | Multimerin 2 | -2.335979473 | 0.014710775 |
| A0A0G2JYL0 | Epas1 | Endothelial PAS domain-containing protein 1 | -3.115395182 | 2.55605E-06 |
| Q5M7T5 | Serpinc1 | Serine (Or cysteine) peptidase inhibitor, clade C (Antithrombin), member 1 | -1.537721938 | 6.81682E-05 |
| F1LWH7 | Tex28 | Testis-expressed 28 | -2.960785402 | 0.0036568 |
| Q6Q0N1 | Cndp2 | Cytosolic non-specific dipeptidase | -1.577839277 | 0.014538714 |
| D4A6W5 | Ndnf | Neuron-derived neurotrophic factor | -1.659246012 | 0.029727063 |
| A0A0G2K151 | Apoe | Apolipoprotein E | -1.485451355 | 0.008067325 |
| A0A0G2JYW6 |  | Uncharacterized protein | -1.357887463 | 0.006544669 |
| O35796 | C1qbp | Complement component 1 Q subcomponent-binding protein, mitochondrial | -1.413066764 | 0.001138444 |
| O35314 | Chgb | Secretogranin-1 | 1.366813599 | 0.003864019 |
| Q4QQV8 | Chmp5 | Charged multivesicular body protein 5 | -1.470718555 | 0.000493895 |
| F7EWC1 | Vasp | Vasodilator-stimulated phosphoprotein | -1.413581349 | 0.009562396 |
| A0A0G2K5J3 | Mfn1 | Mitofusin-1 | 1.339686171 | 0.030364275 |
| Q7TQ16 | Uqcrq | Cytochrome b-c1 complex subunit 8 | 1.393402498 | 0.035630746 |
| P36201 | Crip2 | Cysteine-rich protein 2 | -1.381012519 | 0.000411375 |
| A0A0H2UI05 | Cfap20 | Cilia- and flagella-associated protein 20 | -1.340283023 | 0.004971243 |
| F1LU71 | Auh | AU RNA binding protein/enoyl-coenzyme A hydratase (Predicted), isoform CRA_a | 1.341054391 | 0.003476969 |
| A0A140UHX6 | Sptb | Spectrin beta chain | -1.32198432 | 0.023357611 |
| G3V661 | Baz1b | Bromodomain adjacent to zinc finger domain protein 1B | 1.308734948 | 0.003791505 |
| A0A0G2K330 | Hadhb | Trifunctional enzyme subunit beta, mitochondrial | 1.338563413 | 0.001956434 |
| G3V7K5 | Npc1 | NPC intracellular cholesterol transporter 1 | -1.381215317 | 0.007704527 |
| A0A0G2JV49 | Ppp6r2 | Protein phosphatase 6, regulatory subunit 2 | -1.308201236 | 0.003702224 |
| A0A0G2JV57 | Eif2b1 | Translation initiation factor eIF-2B subunit alpha | -1.37959639 | 0.006620746 |
| M0R6F2 | Acad10 | Acyl-CoA dehydrogenase family, member 10 | 1.309426217 | 0.046839666 |
| D3ZSD3 | Gabpb2 | GA-binding protein transcription factor, beta subunit 2 | 1.411222242 | 0.03757147 |
| D3ZSV1 | Ift20 | Hypothetical LOC287541 (Predicted), isoform CRA_c | -1.300507214 | 0.012941846 |
| F1LNX7 | Tssc1 | Tumor-suppressing subtransferable candidate 1 | -1.313103794 | 0.019169496 |
| F1M7V6 | Cadm4 | Cell adhesion molecule 4 | -1.367416626 | 0.02376085 |
| Q63638 | Speg | Striated muscle-specific serine/threonine-protein kinase | 1.38968178 | 2.98514E-05 |
| D3ZXI2 |  | Uncharacterized protein | -1.32621718 | 0.000595048 |
| Q6AXT8 | Sf3a2 | Splicing factor 3A subunit 2 | -1.4445802 | 0.004976407 |
| D4A2Z6 | Sec63 | SEC63 homolog, protein translocation regulator | 1.417687274 | 2.64553E-05 |
| P00406 | Mtco2 | Cytochrome c oxidase subunit 2 | 1.351171376 | 0.021044475 |
| P85834 | Tufm | Elongation factor Tu, mitochondrial | 1.34208624 | 0.001289246 |
| A0A096MJS4 | Brms1l | Breast cancer metastasis-suppressor 1-like (Fragment) | 1.331147315 | 0.009330663 |
| Q78PB6-2 | Ndel1 | Isoform 2 of Nuclear distribution protein nudE-like 1 | 1.300844652 | 0.018197748 |
| P19969 | Gabra5 | Gamma-aminobutyric acid receptor subunit alpha-5 | 1.31598198 | 0.006348874 |
| G3V6Y9 | Ppig | Peptidyl-prolyl cis-trans isomerase G | 1.319820148 | 0.003389226 |
| B2RYJ1 | Anapc2 | Anapc2 protein | 1.324745366 | 0.009152831 |
| Q9WVK7 | Hadh | Hydroxyacyl-coenzyme A dehydrogenase, mitochondrial | 1.454289212 | 0.001343046 |
| A0A0G2JSQ1 | Sncb | Beta-synuclein | 1.31645589 | 0.000234133 |
| A0A0H2UHP1 | Aldh1a1 | Retinal dehydrogenase 1 | 1.311640288 | 0.001526333 |
| D3ZMY8 | Pcnt | Pericentrin | -1.442203983 | 0.000199513 |
| P09330 | Prps2 | Ribose-phosphate pyrophosphokinase 2 | 1.380903374 | 0.000691854 |
| P11951 | Cox6c2 | Cytochrome c oxidase subunit 6C-2 | 1.378538843 | 0.003463895 |
| P12007 | Ivd | Isovaleryl-CoA dehydrogenase, mitochondrial | 1.318833349 | 0.045365791 |
| B0BNE6 | Ndufs8 | NADH dehydrogenase (Ubiquinone) Fe-S protein 8 (Predicted), isoform CRA_a | 1.362857231 | 0.001776035 |
| D3ZNF4 | Tbl1xr1 | Transducin (Beta)-like 1X-linked receptor 1 (Predicted) | 1.380903841 | 0.018157892 |
| D3ZNY8 | Ror2 | Receptor tyrosine kinase-like orphan receptor 2 | 1.486576264 | 0.041123056 |
| D4A5X7 | Gdap1 | Ganglioside-induced differentiation-associated-protein 1 | 1.323224932 | 0.002167339 |
| F7FKI5 | Pdha1 | Pyruvate dehydrogenase E1 component subunit alpha | 1.307744056 | 0.003194776 |
| Q5U362 | Anxa4 | Annexin | -1.420138653 | 0.008802259 |
| Q5FVJ5 |  | Uncharacterized protein C16orf45 homolog | 1.372256525 | 0.004888417 |
| B0BN72 | Mcrip1 | Mapk-regulated corepressor-interacting protein 1 | -1.336795034 | 0.000787732 |
| Q62770 | Unc13c | Protein unc-13 homolog C | -1.339218562 | 0.015925652 |
| F1M6Z1 | Apob | Apolipoprotein B-100 | -1.456484075 | 0.025971661 |
| A0A0H2UHF6 | Arl6ip5 | PRA1 family protein | -1.329021864 | 0.004927612 |
| P62994-2 | Grb2 | Isoform 2 of Growth factor receptor-bound protein 2 | -1.417691154 | 0.000182572 |
| G3V879 | Coq7 | 5-demethoxyubiquinone hydroxylase, mitochondrial | 1.318438114 | 0.006228364 |
| G3V864 | Plppr4 | Phospholipid phosphatase-related protein type 4 | 1.321968721 | 2.32989E-05 |
| P07150 | Anxa1 | Annexin A1 | -1.343409652 | 0.001739445 |
| A0A0G2JSU1 | Slc1a3 | Amino acid transporter | -1.345617168 | 0.012867883 |
| D3ZHX3 | Diras2 | DIRAS family GTPase 2 | 1.34730476 | 0.013029799 |
| F1M4Q1 | Cylc2 | Cylicin 2 | 1.39331233 | 0.007659247 |
| M0R6L9 | RGD1560687 | Ferritin | -1.314810524 | 0.04582444 |
| Q04940 | Nrgn | Neurogranin | -1.484322708 | 0.001658649 |
| P51653 | Gpc2 | Glypican-2 | 1.325162318 | 0.001597182 |
| Q08013 | Ssr3 | Translocon-associated protein subunit gamma | -1.498171182 | 0.024445982 |
| D3ZBN0 | Hist1h1b | Histone H1.5 | 1.404279037 | 0.000372597 |
| F1M3X5 | Mroh6 | Maestro heat-like repeat family member 6 | 1.47519624 | 0.014055065 |
| Q6P0K8 | Jup | Junction plakoglobin | -1.314092253 | 0.021584979 |
| D3ZRB9 | Tmeff2 | Transmembrane protein with EGF-like and two follistatin-like domains 2 | 1.345203941 | 0.000284365 |
| A1A5N3 | Kcnn4 | Intermediate conductance K channel isoform 4b | 1.386308741 | 0.022275251 |
| D3ZJG4 | Pacs2 | Phosphofurin acidic cluster sorting protein 2 | -1.302100292 | 0.016210422 |
| F1M1K0 | Vwa3a | RCG39867, isoform CRA_b | 1.32009341 | 0.006724434 |
| A0A0G2K5A4 |  | Uncharacterized protein | -1.412661033 | 0.010584237 |
| D4A5B3 | C2cd5 | C2 calcium-dependent domain-containing 5 | 1.411360935 | 0.005249636 |
| A0A096MK08 | Hax1 | HCLS1-associated protein X-1 (Fragment) | 1.387585382 | 0.045585021 |
| P47860 | Pfkp | ATP-dependent 6-phosphofructokinase, platelet type | 1.354699776 | 0.007688344 |
| P47819 | Gfap | Glial fibrillary acidic protein | -1.315953883 | 0.000286544 |
| D3ZXC1 | Itga2b | Integrin subunit alpha 2b | 1.354523788 | 0.002780064 |
| O88752 | Hbe1 | Epsilon 1 globin | 1.309799986 | 0.020466405 |
| E9PU24 | Dnah11 | Dynein, axonemal, heavy chain 11 | 1.325215297 | 0.049244057 |
| Q5PQL2 | Rqcd1 | Cell differentiation protein RCD1 homolog | -1.459663957 | 0.003018918 |
| P43278 | H1f0 | Histone H1.0 | 1.356602326 | 0.000879159 |
| Q6AY55 | Dcakd | Dephospho-CoA kinase domain-containing protein | 1.314451394 | 0.010048031 |
| P22509 | Fbl | rRNA 2'-O-methyltransferase fibrillarin | 1.323891271 | 2.7065E-06 |
| Q62720 | Slc30a1 | Zinc transporter 1 | -1.316503132 | 0.00231573 |
| M0R440 | Zfp326 | Zinc finger protein 326 (Fragment) | 1.397116395 | 0.001242313 |
| D4A197 | Mcee | Methylmalonyl CoA epimerase | -1.330554902 | 0.045888025 |
| D4A1A5 | Ppp2r5c | Protein phosphatase 2, regulatory subunit B', gamma | -1.345839827 | 0.034153614 |
| P24090 | Ahsg | Alpha-2-HS-glycoprotein | 1.390160493 | 0.018533712 |
| G3V9E3 | Cald1 | Caldesmon 1, isoform CRA_b | 1.43287969 | 0.000586898 |
| F1LWV6 | Fam205a | Family with sequence similarity 205, member A | 1.377021648 | 0.023689459 |
| D3ZUC9 | Oxsr1 | Oxidative-stress responsive 1 (Predicted) | -1.301354299 | 0.035720545 |
| A4L9P7 | Pds5a | Sister chromatid cohesion protein PDS5 homolog A | -1.475376053 | 0.000255498 |
| Q9QY02-2 | Ythdc1 | Isoform 2 of YTH domain-containing protein 1 | 1.316102659 | 0.001528555 |
| Nrxn1 | Nrxn1 | Isoform 3 of Neurexin | -3.318333512 | 9.07505E-05 |

**Reference**

MOY, S. S., NADLER, J. J., PEREZ, A., BARBARO, R. P., JOHNS, J. M., MAGNUSON, T. R., PIVEN, J. & CRAWLEY, J. N. 2004. Sociability and preference for social novelty in five inbred strains: an approach to assess autistic-like behavior in mice. *Genes Brain Behav,* 3**,** 287-302.

PRUT, L. & BELZUNG, C. 2003. The open field as a paradigm to measure the effects of drugs on anxiety-like behaviors: a review. *Eur J Pharmacol,* 463**,** 3-33.
